# Propofol administration to the fetal-maternal unit preserved cardiac function in late-preterm lambs subjected to severe prenatal asphyxia and cardiac arrest

**DOI:** 10.1186/2194-7791-1-S1-A1

**Published:** 2014-09-11

**Authors:** Matthias Seehase, Patrick Houthuizen, Reint K Jellema, Jennifer JP Collins, Otto Bekers, Boris W Kramer

**Affiliations:** Department for Pediatric Cardiology and Intensive Care Medicine, Georg-August-University Göttingen, Germany; Dept. of Pediatrics, Maastricht University Medical Center, Maastricht, The Netherlands; Department of Physiology, Cardiovascular Research Institute Maastricht (CARIM), Maastricht University Medical Center, Maastricht, The Netherlands; Department of Clinical Chemistry, Maastricht University Medical Center, Maastricht, The Netherlands

## Aims

Cardiac dysfunction is reported after severe perinatal asphyxia. We hypothesized that maternal propofol anaesthesia during emergency caesarean section diminished cardiac injury in preterm fetuses exposed to global severe asphyxia in utero in comparison to isoflurane anesthesia. We tested if propofol decreased the activity of pro-apoptotic caspase-3 by activating the anti-apoptotic AKT kinase family and the signal transducer and activator of transcription-3 (STAT-3).

## Methods

44 late-preterm lambs underwent standardized umbilical cord occlusion (UCO) or sham-treatment in utero. UCO resulted in global asphyxia and cardiac arrest. Mothers were randomized to either propofol or isoflurane anaesthesia. After emergency caesarean section, the fetuses were resuscitated and anaesthetized for 8h by the anaesthesia of their mothers.

## Results

Propofol treatment resulted 8h after UCO in reduced troponin T levels, in a higher median left ventricular ejection fraction of 84% in comparison to isoflurane (74%), and in reduced activation of caspase-3. Phosphorylated STAT-3 and AKT kinase family were increased to 655% and 500% with propofol after asphyxia.

## Conclusions

Propofol administration preserved cardiac function of late-preterm lambs after asphyxia better than isoflurane. The underlying mechanism may be an activation of the anti-apoptotic STAT-3 and AKT pathway.

